# Left atrial mechanics following left bundle branch area pacing vs. non-conduction system pacing after atrioventricular node ablation in atrial fibrillation

**DOI:** 10.1093/europace/euag116

**Published:** 2026-05-11

**Authors:** Ziad Zalaquett, Roy Chung, Yuichiro Okushi, Joe Demian, Brian Griffin, Pasquale Santangeli, Patrick Collier, Niraj Varma

**Affiliations:** Section of Cardiovascular Imaging, Cardiovascular Medicine, Heart, Vascular, and Thoracic Institute, Cleveland Clinic, Sydell and Arnold Miller Family Heart and Vascular Institute, The Cleveland Clinic Foundation, 9500 Euclid Avenue, Cleveland, OH 44195, USA; Section of Pacing and Cardiac Electrophysiology, Cardiovascular Medicine, Heart, Vascular, and Thoracic Institute, Cleveland Clinic, Cleveland, OH, USA; Section of Cardiovascular Imaging, Cardiovascular Medicine, Heart, Vascular, and Thoracic Institute, Cleveland Clinic, Sydell and Arnold Miller Family Heart and Vascular Institute, The Cleveland Clinic Foundation, 9500 Euclid Avenue, Cleveland, OH 44195, USA; Section of Pacing and Cardiac Electrophysiology, Cardiovascular Medicine, Heart, Vascular, and Thoracic Institute, Cleveland Clinic, Cleveland, OH, USA; Section of Cardiovascular Imaging, Cardiovascular Medicine, Heart, Vascular, and Thoracic Institute, Cleveland Clinic, Sydell and Arnold Miller Family Heart and Vascular Institute, The Cleveland Clinic Foundation, 9500 Euclid Avenue, Cleveland, OH 44195, USA; Section of Pacing and Cardiac Electrophysiology, Cardiovascular Medicine, Heart, Vascular, and Thoracic Institute, Cleveland Clinic, Cleveland, OH, USA; Section of Cardiovascular Imaging, Cardiovascular Medicine, Heart, Vascular, and Thoracic Institute, Cleveland Clinic, Sydell and Arnold Miller Family Heart and Vascular Institute, The Cleveland Clinic Foundation, 9500 Euclid Avenue, Cleveland, OH 44195, USA; Section of Pacing and Cardiac Electrophysiology, Cardiovascular Medicine, Heart, Vascular, and Thoracic Institute, Cleveland Clinic, Cleveland, OH, USA

**Keywords:** Left bundle branch area pacing, Atrial fibrillation, Nodal ablation, Speckle-tracking echocardiography

Atrioventricular nodal (AVN) ablation with ventricular pacing for rate control benefits patients with atrial fibrillation (AF) and rapid ventricular response.^1^ Pacing mode matters. Right ventricular pacing (RVP) may result in heart failure by forcing ventricular dyssynchrony.^2^ In contrast, biventricular pacing (BiV) post-AVN ablation improved survival.^3^ Left bundle branch area pacing (LBBAP) may confer additional benefit, as it restores normal sinus rhythm (NSR) in some ‘refractory’ AF patients undergoing AVN ablation.^4^ The mechanism underlying this phenomenon is incompletely understood. We hypothesized that changes in left atrial (LA) function would accompany ventricular pacing in AF patients following AVN ablation, and the direction of these changes would differ between LBBAP and non-conduction system pacing (non-CSP).

We retrospectively analysed patients with refractory, uncontrolled AF who underwent AVN ablation and either LBBAP or non-CSP (RVP, leadless, or BiV) with interpretable pre- and post-procedural echocardiograms during follow-up (2019–23) at the Cleveland Clinic. Demographic and clinical characteristics were extracted from electronic medical records. The study received IRB approval.

LBBAP technique followed standard procedure using lumenless leads as previously described^5^ [left ventricle (LV) activation time of <80 ms with unipolar pacing, RBBB pattern in V1, interpeak interval >33 ms and presence of LBB potential]. Non-CSP included RVP (apical or septal) and BiV (coronary sinus lead with an RV lead).

The primary objective was to compare the impact of LBBAP vs. non-CSP on echocardiographic measures of LA function. Standard volumetric and functional parameters were assessed pre- and post-procedure. LA maximal and minimal volumes were measured and indexed to body surface area, and LA emptying fraction calculated accordingly. LA strain [reservoir (LASr), conduit (LAScd), and contractile (LASct)] was assessed from apical four-chamber views and averaged over three consecutive cycles in AF, and LV global longitudinal strain (GLS) from apical two-, three-, and four-chamber views using TomTec software, with manual adjustment as needed. All measurements demonstrated excellent intra- and inter-observer reliability (intra-class correlation coefficient >0.80). Spontaneous occurrence of NSR was assessed during follow-up (mean 2.7 ± 1.5 years).

Normality of continuous variables was assessed using the Shapiro–Wilk test. Pre- and post-pacing comparisons were performed using paired *t*-tests. The effect of LBBAP vs. non-CSP on post-pacing echocardiographic parameters was evaluated using analysis of covariance (ANCOVA) with adjustment for baseline values and relevant covariates. A *P*-value of <0.05 was considered statistically significant.

The study cohort comprised 73 patients [26% male; age 79.3 ± 7.4 years; left ventricular ejection fraction (LVEF) 53.5 ± 14%]. Compared with non-CSP patients (*n* = 38), those treated with LBBAP (*n* = 35) had a higher prevalence of coronary artery disease (32% vs. 9%), chronic kidney disease (47% vs. 20%), and prior pulmonary vein isolation (40% vs. 16%) but baseline LA volumes were smaller (71 vs. 85 mL). Baseline LV GLS was similar between groups. Baseline QRS duration did not differ between non-CSP and LBBAP groups (102.1 ± 30.1 vs. 91.7 ± 21.8 ms). Among non-CSP patients, 18 (47.4%) underwent BiV pacing, 16 (42.1%) RV pacing, and 4 (10.5%) leadless pacing.

Overall, mean heart rate decreased post-procedure (89 vs. 77 bpm, *P* < 0.001) (baseline and post-procedural paced heart rates were similar between groups). LVEF remained unchanged post-procedure while LV GLS significantly improved (−13.3% vs. −11.5%, *P* = 0.008). In LBBAP, paced QRS duration was significantly narrower (115.6 vs. 141.2 ms, *P* < 0.001). Among LBBAP patients, median RWPT-V6 was 70 ms (IQR 65–80) and mean V1–V6 interpeak interval was 38.7 ± 8.9 ms. The interval between pre- and post-pacing echocardiograms did not differ between LBBAP and non-CSP groups (15.6 ± 11.8 vs. 18.4 ± 11.3 months).

In LBBAP, absolute values of LASr (11.5% vs. 14.5%, *P* = 0.004), LAScd (−7.5% vs. −11.4%, *P* = 0.002), and LV GLS (−11.8% vs. −14.6%, *P* = 0.01) significantly increased after pacing (*Figure 1A*). In contrast, non-CSP pacing did not affect LASr or LAScd but reduced LASct (−3.5% vs. −1.5%, *P* = 0.02). Compared with non-CSP, LBBAP resulted in greater improvements in LASr (adjusted mean difference = +3.9, *P* = 0.001), LASct (adjusted mean difference = −2.1, *P* = 0.02), and LV GLS [adjusted mean difference = −2.2, *P* = 0.02 (*Figure 1B*)] (ANCOVA). This was accompanied by lower indexed LA minimal volume (adjusted mean difference = −8.7, *P* = 0.02) and maximal volume (adjusted mean difference = −11.9, *P* = 0.002) compared to non-CSP (*Figure 1B*).

**Figure 1:**
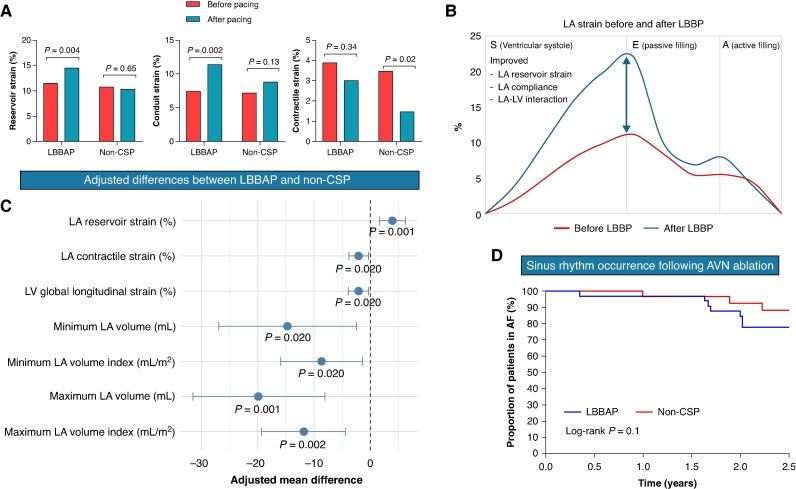
(*A*) changes in LA strain parameters before and after pacing in patients undergoing LBBAP compared with non-CSP. (*B*) Representative example illustrating improvement in LA strain curves in the same patient before and after LBBAP. Arrow shows increased LA reservoir strain which translates to improved LA compliance and LA–LV interaction. (*C*) Echocardiographic parameters demonstrating significant adjusted differences between the LBBAP and non-CSP groups, with non-CSP used as the reference category. (*D*) Kaplan–Meier curves demonstrating a higher incidence of return to sinus rhythm in patients undergoing LBBAP compared with non-CSP during follow-up.

Spontaneous restoration of NSR occurred in 9/35 (26%) LBBAP patients vs. 3/38 (8%) non-CSP patients (*P* = 0.06) during a mean follow-up of 2.7 years. Among LBBAP patients who reverted to NSR, LASr increased by 3.8%, LAScd by 4.6%, and LASct by 1.6%, with reductions in indexed minimal and maximal LA volumes (11.5 and 11.6 mL, respectively). LVEF increased by 2.5%, and LV GLS improved by 1.7% on average.

Our results indicate that LBBAP significantly improved LA mechanics, reflected by increased reservoir and contractile strain with lower LA volumes, improved LV function, and a higher rate of conversion to NSR compared with non-CSP, in patients without significant LV dysfunction.

LA function is linked to the development and persistence of AF and is modulated by LV mechanics. LA stretch plays an important role in AF development in patients with dual-chamber pacemakers,^6^ and lower LA contractile strain and higher LA volumes have been independently associated with incident AF.^7^ LV diastolic recoil, itself a function of LV contractility, facilitates LA emptying by reducing LA distending forces. Hence, LBBAP, by maintaining or enhancing LV function (LV GLS improved in our study) may promote better LA emptying than RVP, which induces LV dyssynchrony and may acutely increase LA distention and AF burden.^8^ This mechanism may explain the trend towards higher spontaneous reversion to NSR with LBBAP. Notably, this was observed despite nearly 50% of non-CSP patients receiving BiV pacing which mitigates RVP’s deleterious effects^3^ and may yield outcomes comparable to CSP in some settings.^9^ Similarly, others have reported a lower incidence of AF with LBBAP compared with BiV.^10^ Our results are remarkable since our patients had ‘permanent’ AF, some with ineffective prior PVI, likely with significant atrial remodelling and fibrosis, and considered to have irreversible arrhythmia. This points to the influence of LV—and thereby LA—mechanical function in atrial arrhythmogenesis. The habit of not implanting an atrial lead may need to be carefully considered.

Our study is limited by sample size, non-randomized groups and use of various non-CSP pacing modalities. However, the results signal the importance of choice of pacing on LV and LA mechanics and AF progression—or reversion.

## Data Availability

The data underlying this article will be shared on reasonable request to the corresponding author.
